# Malignant Peripheral Nerve Sheath Tumors—A Comprehensive Review of Pathophysiology, Diagnosis, and Multidisciplinary Management

**DOI:** 10.3390/children9010038

**Published:** 2022-01-01

**Authors:** Samantha W. E. Knight, Tristan E. Knight, Teresa Santiago, Andrew J. Murphy, Abdelhafeez H. Abdelhafeez

**Affiliations:** 1 Division of Surgery, Department of General Surgery, Southern Illinois University School of Medicine, Springfield, IL 62702, USA; sknight55@siumed.edu; 2 Cancer and Blood Disorders Center, Seattle Children’s Hospital, Seattle, WA 98195, USA; knight.tristan@gmail.com; 3 Division of Hematology and Oncology, Department of Pediatrics, University of Washington School of Medicine, Seattle, WA 98195, USA; 4 Department of Pathology, St. Jude Children’s Research Hospital, Memphis, TN 38105, USA; Teresa.Santiago@STJUDE.ORG; 5 Department of Surgery, St. Jude Children’s Research Hospital, Memphis, TN 38105, USA; andrew.murphy@stjude.org; 6 Division of Pediatric Surgery, Department of Surgery, University of Tennessee Health Science Center, Memphis, TN 38105, USA

**Keywords:** malignant peripheral nerve sheath tumor, MEK inhibitor, multi-disciplinary management, neurofibroma, neurofibromatosis type 1

## Abstract

Malignant peripheral nerve sheath tumors (MPNSTs) are aggressive soft tissue sarcomas (STS) with nerve sheath differentiation and a tendency to metastasize. Although occurring at an incidence of 0.001% in the general population, they are relatively common in individuals with neurofibromatosis type 1 (NF1), for whom the lifetime risk approaches 10%. The staging of MPNSTs is complicated and requires close multi-disciplinary collaboration. Their primary management is most often surgical in nature, with non-surgical modalities playing a supportive, necessary role, particularly in metastatic, invasive, or widespread disease. We, therefore, sought to provide a comprehensive review of the relevant literature describing the characteristics of these tumors, their pathophysiology and risk factors, their diagnosis, and their multi-disciplinary treatment. A close partnership between surgical and medical oncologists is therefore necessary. Advances in the molecular characterization of these tumors have also begun to allow the integration of targeted RAS/RAF/MEK/ERK pathway inhibitors into MPNST management.

## 1. Introduction

Malignant peripheral nerve sheath tumors (MPNSTs) are malignant, locally aggressive soft tissue sarcomas (STS) with nerve sheath differentiation and a high propensity to metastasize. They are rare in the general population, with an approximate lifetime incidence of 0.001% (e.g., 1/100,000) [1]. However, in individuals with neurofibromatosis type 1 (NF1), the lifetime risk of developing one of these tumors is approximately 10%. Up to 50% of all MPNSTs occur in patients with NF1 [1]. All-told, MPNST comprise 5–10% of soft tissue sarcomas and are one of the most common nonrhabdomyosarcomatous soft tissue sarcomas (NRSTS) in pediatric patients [1]. Whereas about 10–20% of all MPNSTs are diagnosed in children [1], there is no difference between children and adults in tumor location, size, or histological grade–although adults are more likely to have more than one primary tumor at the time of diagnosis [1].

MPNSTs may arise de novo from a peripheral nerve ORAs a malignant transformation of a pre-existing benign nerve sheath tumor–especially neurofibromas [2]. They may occur anywhere in the body but are most often axial in location and are diagnosed based on histopathological-demonstrated peripheral nerve sheath differentiation [2].

## 2. Malignant Peripheral Nerve Sheath Tumors in Context as Soft Tissue Sarcomas

MPNSTs are a form of sarcoma–that is, a tumor arising from cells of mesenchymal origin that have undergone malignant transformation. Mesenchymal cells display at least partial differentiation towards a connective tissue lineage–a broad term that includes, among others, muscle, adipose, bone, cartilage, vascular, and nervous tissue. Sarcomas are therefore classified based on the specific type of mesenchymal tissue they have arisen from and/or the tissue to which they bear a histopathological resemblance. Sarcomas are broadly divided into either soft tissue or bony tissue. Soft tissue sarcomas (STS) together comprise 7% of pediatric solid tumors [3] and are divided into those which either display or do not display differentiation towards striated muscle: rhabdomyosarcomas and nonrhabdomyosarcomatous soft tissue sarcomas (NRSTS), respectively. The category of NRSTS contains a diverse array of sarcomas, as any STS which does not display striated-muscle differentiation is necessarily included in this broad grouping.

According to the Fifth Edition of the World Health Organization (WHO)’s Classification of Tumors Soft Tissue and Bone Tumors, [4] nerve sheath tumors are divided into those which are benign (e.g., schwannomas, neurofibromas including plexiform neurofibromas, perineuriomas, etc.) and those which are malignant, of which MPNSTs form a major subset [5]. Although sometimes used synonymously with the terms “malignant schwannoma” or “neurofibrosarcoma,” MPNST is the most accurate moniker, as these tumors may originate from and/or display differentiation towards any peripheral nerve sheath cell–not only Schwann cells [2]. 

## 3. MPNST Pathophysiology

Specific NRSTS may occur more commonly within the context of particular cancer predisposition syndromes (e.g., leiomyosarcoma in hereditary/germline retinoblastoma, due to *RB1* mutation) [6]. Alternatively, certain NRSTS may be part of the diagnostic criteria for a given disorder (e.g., rhabdoid tumors in rhabdoid tumor predisposition syndrome, due to *SMARCB1/INI1* mutation) [7]. MPNSTs are the former, as their presence does not specifically define the presence of Neurofibromatosis type 1 (NF1), nor are they required for its diagnosis. Still, they are considered a hallmark of NF1 when present. Similarly, many NRSTS are characterized by specific chromosomal translocations or mutations. The presence of such changes typically has one of two results. Firstly, a fusion protein may be generated, allowing for activation of a constitutively expressed kinase or transcription factor independent of ligand binding [8]. Secondly, the mutation may cause a deleterious loss-of-function in a tumor suppressor or cell-cycle regulator gene [9]. These alterations are detectable via polymerase chain reaction (PCR), and their presence helps to facilitate diagnosis within this heterogeneous group of tumors. MPNSTS are most often characterized by the second variety of mutations–specifically by loss of function of the tumor suppressor gene NF1. 

Although bi-allelic NF1 inactivation or mutation appears necessary for MPNST development, it does not seem sufficient [10]. Plexiform neurofibromas were shown to develop in this context [11], but malignant transformation appears to require additional abnormalities–specifically, CDKN2A, EGFR, SUZ12, and TP53 have all been implicated [12]. *EGFR*, *SUZ12*, and *TP53* mutations are all seen in the context of MPNSTs, but not in plexiform neurofibromas or atypical neurofibromas [12]. Similarly, CDKN2A loss is seen in the vast majority of atypical neurofibromas and in low-grade MPNSTs–but not in plexiform neurofibromas [13]. Therefore, one potential model for MPNST development proposes that bi-allelic NF1 loss occurs in nerve-sheath precursor cells, resulting in benign neurofibroma formation. Subsequently, loss of CDKN2A occurs, promoting the development of an atypical neurofibroma–followed by additional mutations in *EGFR*, *SUZ12*, and/or *TP53,* which cause transformation into an MPNST [10].

### Relationship of MPNST to Neurofibromatosis Type 1

NF1 is a neurocutaneous cancer predisposition syndrome, which may arise either *de novo* or be inherited in an autosomal dominant fashion. A diagnosis of NF1 may be established when an individual meets the National Institutes of Health diagnostic criteria (provided in Table 1). It is characterized by deleterious alterations in the NF1 tumor suppressor gene at 17q11.2, resulting in heterozygous, loss-of-function mutations. Therefore, the production and/or function of its gene product, the protein neurofibromin, is subsequently impaired [14]. This protein interacts with several key cellular pathways and has multiple functions–the most germane of which is its role as a critical tumor suppressor gene via negative regulation of the RAS/RAF/MEK/ERK pathway [9,15]. Loss-of-function mutations in *NF1,* therefore, enable constitutive activation of this pathway, with resultant cell growth and proliferation. Patients with NF1 possess only a single functional copy of the NF1 gene (e.g., the “first hit”), with loss of the second copy acting as an oncogenic “second hit” and thereby allowing constitutive activation of this pathway [9]. Patients with NF1 are therefore at increased risk for multiple neoplastic processes–including MPNSTs [14]. A particular characteristic of NF1 is the extreme heterogeneity of its clinical manifestations, which may vary wildly even among members of the same kindred [16]. No specific alteration of the NF1 gene is specifically associated with the NF1 syndrome, and over 500 discrete mutations were identified [16]. This variability may be due both to the large size of the NF1 gene, which lends itself to a greater frequency of mutations, as well as the large number of possible means in which the function or amount of neurofibromin produced may be affected [16]. Genotype-phenotype correlations do exist for some specific mutations, but these constitute the minority of cases [17]. 

Though their presence is a hallmark of NF1, the presence of an MPNST does not ipso facto indicate a diagnosis of NF1. Although the most common neoplasms seen in patients with NF1 are benign neurofibromas, MPNSTs are the most common *malignant* neoplasm in this population, occurring in approximately 10% of patients with NF1 [16]. Conversely, as many as half of all MPNSTs are seen in patients with NF1 [1]. Although many MPNSTs are therefore sporadic, a diagnosis of NF1 is the primary known risk factor. Moreover, among those patients with NF1, a family history of NF1 and MPNST appears to be associated with an approximately three-fold greater risk of developing an MPNST in that patient [18]. Patients with whole-gene deletions of NF1, subcutaneous neurofibromas, or a larger number of plexiform neurofibromas are at particular risk of developing MPNST [19,20]. Compared to patients with sporadic MPNSTs, patients with NF1-associated MPNSTs also present them at an earlier age–typically in the second-to-fourth decades of life [21]. Besides a diagnosis of NF1, the other primary known risk factor for MPNST development is radiation exposure, typically in the context of a secondary malignant neoplasm occurring following radiotherapy [22]. 

In most series, patients with either a diagnosis of NF1 or prior radiotherapy have shown a worse overall survival compared to those with sporadic MPNSTs—likely due to the greater propensity towards metastases and/or local invasion demonstrated by tumors in these patients [22,23,24]. However, the presence of NF1 itself does not directly appear to be the causative risk factor for these poorer outcomes–instead, patients with NF1 tend to have larger tumors, which are more challenging to fully resect [25]. The survival gap does appear to be narrowing, however, with patients with NF1-related MPNSTs faring better in studies performed more recently—though still not as well as those with sporadic MPNSTs [23].

## 4. Characteristics of MPNSTs And Differentiation from Plexiform Neurofibromas

### 4.1. Clinical Features

Patients with MPNSTs typically present with a history of a progressively expanding soft-tissue mass, which may or may not be painful [2]. In particular, the development of new neurological symptoms (e.g., hypoesthesia or dysesthesia), pain, and/or enlargement of an existing plexiform neurofibroma should raise suspicion for malignant transformation into an MPNST [26]. Symptoms are otherwise relatively non-specific and may be related to the disease site, e.g., neurologic compromise in the event of invasion into a nerve plexus or mass-effect due to tumor size/location [2].

### 4.2. Radiology

**Figure 1:**
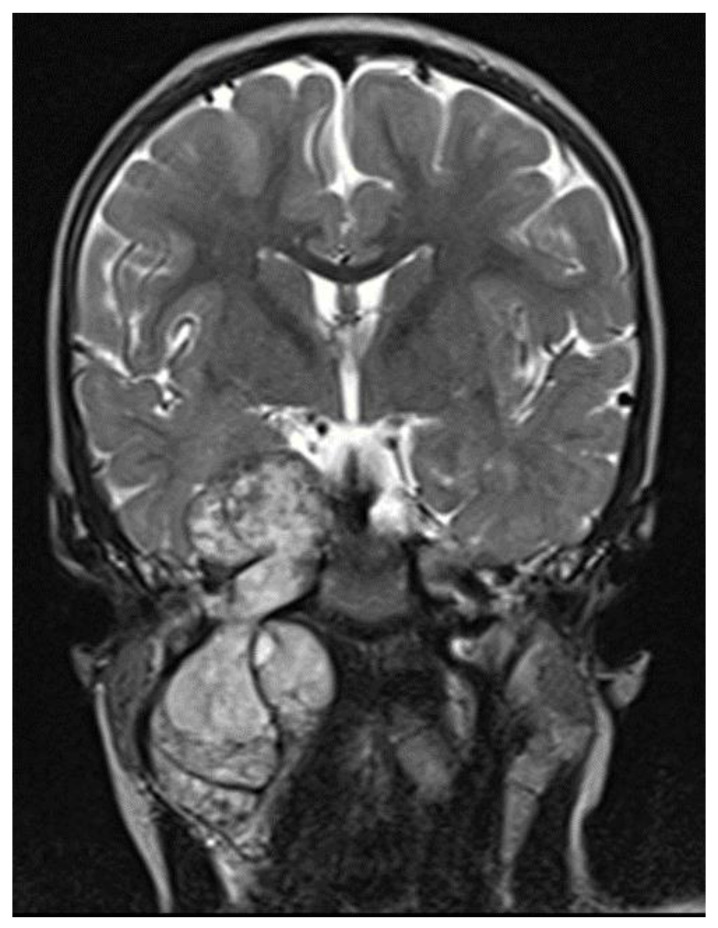
MRI T2 images, intratumor heterogeneity.

**Figure 2:**
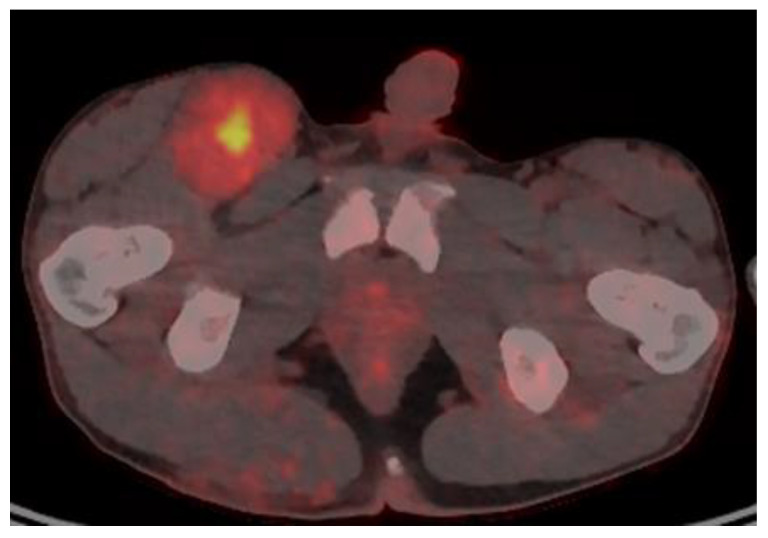
18F-FDG PET/CT of a patient with NF1 and an MPNST of the right groin, with a maximal SUV of 7.

Imaging studies are necessary to delineate tumor extent and may also be of some use in differentiating MPNSTs versus plexiform neurofibromas. On MRI, features such as surrounding peritumoral edema, irregular and/or locally invasive margins, and intra-tumoral heterogeneity appear to be more indicative of MPNSTs (See Figure 1). However, the reported sensitivity and specificity of such findings are quite variable–ranging from less than 20% to over 90% [27,28,29]. Because MPNSTs exhibit higher metabolic activity than plexiform neurofibromas, 18F-FDG PET/CT may be useful in discriminating between these two entities (See Figure 2). Although some degree of overlap exists, standard uptake values (SUVs) of 1–4 tend to indicate benign tumors, while SUVs of 3–21 suggest the presence of MPNST [30,31,32]. Depending on the threshold used, sensitivity and specificity are greater than 90% and 70%, respectively, for the detection of MPNSTs [30,31,32]. A tumor SUV of greater than 1.5-times that of normal hepatic tissue was also separately shown to be both a sensitive and specific indicator of MPNSTs [33].

### 4.3. Histopathology

Although clinical and radiological features may be suggestive, definitive diagnosis of an MPNST requires histologic examination. This is complicated, however, by the absence of specific pathognomonic histopathological features, and diagnosis may be challenging [34]. A clear origin from either a peripheral nerve or a neurofibroma strongly aids in diagnosis [2]. Additionally, specific histologic features should be present, including fascicles with alternating, marble-like cellularity, palisade/rosette-like arrangements, and asymmetric spindle cells [2]. Based on the presence of high amounts of mitotic figures and necrosis, MPNSTs may be classified as high-grade or, conversely in the absence of necrosis/fewer mitotic figures, may be classified as low-grade (Figure 2).

**Figure 3:**
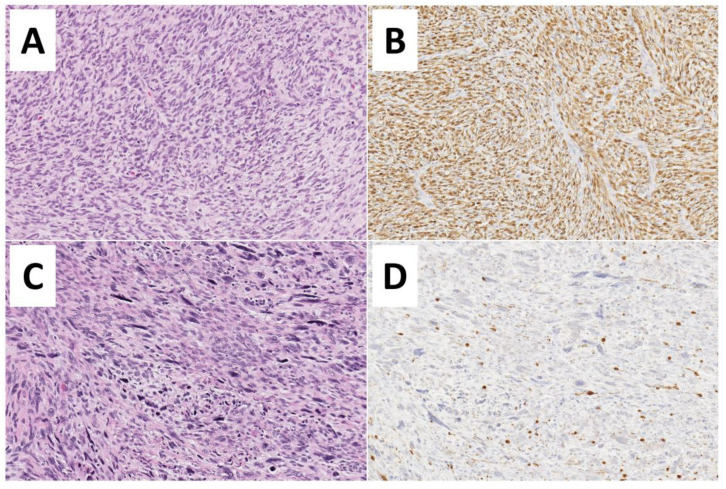
Histopathological features of Malignant Peripheral Nerve Sheath Tumors—MPNSTs. (**A**,**B**) Low-grade MPNST composed of a spindle cell proliferation with increased cellularity, mild cytological atypia, few mitoses (3 to 9 mitoses per 10 high-power fields), but no evidence of necrosis (Hematoxylin and Eosin, 200×). Diffuse immunoreactivity for S100-protein is noted (200×). (**C**,**D**) High-grade MPNST showing marked hypercellularity, nuclear pleomorphism, necrosis, and numerous mitoses (more than 10 mitoses per 10 high-power fields) (Hematoxylin and Eosin, 200×). Only focal S100-protein positivity is observed.

Low-grade MPNSTs are sometimes difficult to histologically differentiate from benign plexiform neurofibromas [35]. Moreover, multiple patterns may exist in a single tumor, necessitating careful and complete examination [35]. A biopsy specimen may be insufficient to characterize the tumor adequately, and resection is preferred where possible [10]. However, in cases where the primary tumor is unresectable, percutaneous image-guided core-needle biopsy was shown to be highly accurate in differentiating benign versus malignant peripheral nerve sheath tumors [36] and may therefore be a reasonable option. The need to obtain adequate tissue for examination is counterbalanced by the potential for nerve injury. The risk of neurological deficits was reported as being as high as 60% in some series [37] and entirely absent in others [36]. This discrepancy likely arises secondary to the greater precision allowed by more recent diagnostic techniques, specifically, image guidance [36]. Immunohistochemical staining may include S100, Ki67, TP53, CD34, p16, and H3K27me3 (trimethylation at lysine 27 of histone H3) [35]. The interested reader is referred to an excellent review article on the histopathological diagnosis of nerve sheath tumors in general, including MPNSTs [38], see Figure 3.

## 5. Staging and Risk Group Assignment of MPNST

There is no disease-specific staging system for MPNST. Rather, MPNSTs (and NRSTS) were staged using two separate systems: The Intergroup Rhabdomyosarcoma Study Staging System [39,40] and the American Joint Committee on Cancer (AJCC) TNM staging system [41]. The former system was utilized for earlier trials, with the adoption of the AJCC staging system in more recent studies [42]. The recent Children’s Oncology Group (COG) ARST0332 trial (which included MPNSTs) utilized the AJCC sixth edition staging system, which was current at the time of study inception. The eighth edition of these guidelines was recently published [43]. Briefly, the AJCC staging system utilizes the size, depth, and invasiveness of the tumor (T), the presence/absence of lymph node involvement (N), and the presence/absence of distant metastasis (M) to assign a stage from 1 to 4, some of which include multiple sub-stages. In addition, tumor histologic grade is a key consideration–and is included in both the eighth edition of the AJCC staging system and ARST0332 risk group assignments [42,43]. Both the Federation Nationale des Centres de Lutte Contre le Cancer (FNCLCC) [44] and Pediatric Oncology Group (POG) [45] assign tumors to either a low-grade (POG 1 or 2, FNCLCC 1) or high-grade (POG 3, FNCLCC 1 or 2), on the basis of tumor differentiation, mitosis, and necrosis. Both systems are prognostic, with high-grade predicting a poorer outcome [42,46]. 

Modern treatment strategies for NRSTS in general (including MPNSTs) are therefore ultimately based on that tumor’s local characteristics, nodal involvement, metastatic spread, and histologic grade–as well as the extent of surgery/margin status [42]. From these, a stage and risk group may be assigned, which informs subsequent management. The COG ARST0332 trial assigned patients to one of three risk groups. Low-risk tumors were defined as: non-metastatic, low-grade, grossly resected tumors irrespective of margin status, OR non-metastatic, high-grade, grossly resected tumors regardless of margin status and ≤5 cm. Intermediate-risk tumors were defined as non-metastatic, high-grade, grossly resected tumors irrespective of margin status and >5 cm, OR non-metastatic, unresected tumor of any size or grade. Finally, high-risk tumors were defined as those with metastases to lymph nodes, distant sites, or both [42]. Based on risk group assignment, treatment groups were assigned; these are outlined below. Therefore, it is reasonable to stage MPNSTs using this strategy, as the ARST0332 clinical trial will likely inform future management and studies of NRSTs, including MPNSTs.

## 6. Management of MPNSTs

As a group, NRSTS may be crudely divided into those which are more sensitive to chemotherapy (e.g., synovial sarcoma [47]) and those which are less sensitive (e.g., clear cell sarcoma [48]). In either case, the most critical component of management is surgical resection–particularly in the latter group. MPNSTs are poorly responsive to chemotherapy, at least partially due to their slow growth rate. Their primary management is therefore surgical in nature, with non-surgical modalities playing a supportive yet essential role, particularly in metastatic, invasive, or widespread disease.

Patients with MPNSTs were eligible for inclusion in the recently reported COG ARST0332 trial [42]. Fifty-eight patients with MPNSTs were included, comprising 11% of the total study population (N = 529, and constituting the second-largest cohort after those with synovial sarcoma (25%; N = 128). Following risk group assignment (as outlined above), patients were enrolled into one of four treatment groups: surgery alone (Arm A), surgery plus adjuvant radiotherapy (Arm B), surgery plus adjuvant chemotherapy and radiotherapy (Arm C), or neoadjuvant chemotherapy and radiotherapy, surgery, and then adjuvant chemotherapy and radiation therapy (Arm D). Both Arm C and Arm D utilized combined doxorubicin plus ifosfamide chemotherapy. Patients with MPNSTs were represented in each of the four treatment groups and were assigned as follows: 7 patients on Arm A, 2 on Arm B, and 19 patients on Arm C. Most patients with MPNSTs were enrolled on Arm D (*n* = 32). Although MPNST-specific results are not available at this time, general outcomes are compared favorably to prior studies. At a median follow-up time of 6.5 years, that study’s 5-year overall survival (OS) and event-free survival (EFS) were as follows—low risk: 96.2% and 88.9%, respectively. Intermediate risk: 79.2% and 65.0%, respectively. High risk: 35.5% and 21.2%, respectively [42].

Given MPNSTs’ locally aggressive nature and tendency to metastasize, multidisciplinary, multi-modality management is frequently a feature of many patients’ treatment courses. It is, therefore, somewhat artificial to discuss each in isolation. Additionally, patients with more advanced disease are more likely to both receive more intensive/multi-modality therapy–and to have worse outcomes. We have, however, sought to provide an overview of unique, modality-specific considerations below.

### 6.1. Surgical Management and Surgery-Specific Considerations

Resectability is strongly related to outcomes, and survival depends upon wide resection [49,50]. Wide local resection involving R0 resection with a least 2 cm margin in all directions is a major determinant of outcomes in patients with soft tissue sarcomas in general [51,52]. However, in patients with MPNST, R0 resection is not always possible due to the nature and biology of these tumors.

MPNST can develop anywhere in the body but occur mainly at the location of major nerve plexuses/roots. Extremities are most commonly involved, followed by the trunk, and head, and neck. MPNSTs of the extremities have superior outcomes compared to other disease sites, most likely secondary to an enhanced ability to achieve R0 resections and improved tumor control [49]. The most common extremity location for MPNST is the brachial plexus, with total resection being the goal. Here, and in other areas, R0 resection may result in unacceptable morbidity. Historically, treatment usually included local resection to confirm the diagnosis. This was followed by amputation for more proximal involvement of the associated plexus–resulting in severe neurological deficits. If the relevant local vasculature was also involved, the resulting resection would also compromise local blood flow and potentially result in limb ischemia/necrosis. If an en bloc resection of a major nerve bundle were performed, such as the brachial plexus, sciatic nerve, or spinal roots in the trunk, this would also create circumstances leading to the development of pressure sores and necessitating secondary amputations. 

Conversely, current literature supports limb-sparing procedures (LSP) [49,50,52]. LSPs allow for retention of the afflicted limb in the context of localized disease [49,53]. Limb sacrifice (e.g., amputation) therefore occurs in approximately 5–10% of patients for whom wide excision is not feasible–and who can tolerate such surgery and do not have metastatic disease [49,53]. Those who do undergo LSP also undergo local soft tissue irradiation with implanted rods supplemented by external beam radiation. When contemplating whether to perform limb-sparing versus limb-sacrifice procedures, it is important to consider that for MPNSTs at the level of the plexus, amputation offers superior survival as compared to local resection if an R0 resection is not possible [49,54].

In patients with head and neck MPNSTs, R0 resection is extremely difficult to achieve secondary to local anatomy and critical structures. This likely contributes to the poorer outcome seen in these patients. Wide local resection and R0 margins remain the goal, however. If not possible, resection of the tumor without sacrificing major neurovascular structures is appropriate, followed by adjuvant therapy [52].

Intraoperatively, the surgeon may appreciate a firm, solid mass. Locally advanced disease with invasion to the adjacent tissue or vasculature is common. There may also be disruption of anatomical planes with edema. The surgeon may perform intraoperative high-resolution ultrasound, which may reveal an irregular, isoechoic, or hypo to hyperechoic structure with solid and cystic structures. On ultrasound and on gross pathology, features of necrosis may be present [55].

Some literature would suggest that patients with localized soft tissue sarcomas may benefit from undergoing re-resection if there was an apparent macroscopic tumor left in the tumor bed. This approach decreases local tumor burden in anticipation of or following local radiotherapy [52,56]. Given the rarity of these tumors, their aggressive nature, and the relative lack of literature, further studies are needed to fully understand the optimal surgical management of these tumors.

### 6.2. Traditional Chemotherapy

Prior to the COG trial ARST0332, a small number of studies have evaluated MPNST responses to traditional chemotherapeutic agents–typically mono-or-combination therapy with ifosfamide and/or anthracyclines (doxorubicin/epirubicin), sometimes in combination with etoposide. These studies assessed both neoadjuvant [57,58,59] and adjuvant [60,61,62] approaches. Because of the relative rarity of NRSTS of all subtypes, randomized controlled clinical trials are lacking, and it is difficult to draw definitive conclusions from the existent studies. Although considerable inter-study heterogeneity exists, systemic chemotherapy has generally been administered in the setting of locally invasive, large (e.g., >5 cm), deeply located, and/or metastatic disease. Unsurprisingly response rates are disappointing, generally clustering at approximately 50%, with the vast majority of these being either stable disease or partial response [63]. 

Patients with MPNST have also occasionally been included in studies of other soft-tissue sarcomas. In those studies, they have generally been treated as-per rhabdomyosarcoma protocols–with primary excision where possible, or neoadjuvant chemotherapy if not, and adjuvant chemo/radiotherapy for those at risk of local reoccurrence [64]. Agents administered are typical of rhabdomyosarcoma therapy and overall response rates for patients requiring chemotherapy are generally also around the 50% mark–though regimens utilizing ifosfamide achieved markedly higher response rates [64]. However, any interpretation of such studies must be under the caveat that staging/grading of MPNSTs is not consistent across trials, which complicates comparison.

### 6.3. Targeted and Novel Agents

Partially because MPNSTs are resistant to traditional chemotherapeutic agents, the use of targeted therapies is appealing. These strategies are based upon the improving understanding of the molecular basis of this neoplasm. As outlined above, MPNSTs possess multiple targetable pathways [13,65]. Of these, the most implicated appears to be the Ras/Raf/MEK/ERK pathway [65]. This pathway is also relatively well understood and one against which multiple inhibitors exist. A complete response to MEK inhibition was reported [66], and within the context of MPNST, clinical trials of Ras/Raf/MEK/ERK pathway inhibition are ongoing [67]. This approach has also seen relatively well-documented success in other NF1-associated tumors, including low-grade gliomas (LGGs) and plexiform neurofibromas [15,68], with formal clinical trial assessment underway [69,70,71]. As is the case with MPNSTs, these tumors may be poorly responsive or refractory to “traditional” chemotherapeutic management. The interested reader is referred to an excellent recent review article on potential novel pharmacologic approaches to the management of peripheral nerve sheath tumors-including existent clinical trials [63]. It should be reiterated that such practices remain experimental, and patients should be enrolled in clinical trials to better understand the efficacy of these therapies.

Moreover, essentially all-such trials require these patients’ MPNSTS to be partially or unresectable, inoperable, metastatic, or otherwise not amenable to definitive surgical management. Finally, the optimal strategies for integrating surgical resection with targeted therapies remain to be developed. We speculate, however, that neoadjuvant pharmacological therapy could best be used to render unresectable tumors operable–essentially recapitulating the successful strategies utilized in more chemo-sensitive NRSTS, but with pathway-specific agents. 

The field of pediatric oncology is rife with instances of rationally designed, targeted therapies achieving breakthroughs and becoming standard of care/frontline therapy–such as was achieved via the use of the anti-GD2 antibody dinutuximab for patients with high-risk neuroblastoma [72]. However, it is at least as common, if not more so, for mechanistically promising therapies to fail upon clinical translation [73,74]. Therefore, at the present time, complete surgical resection is the only therapy that provides a durable cure for MPNSTs. Within the next decade, however, it would not be surprising to witness the integration of standardized pharmacological neoadjuvant or targeted adjuvant therapies, with definitive surgical management–not only for tumors that are metastatic or non-resectable but as a matter of course to minimize pre-operative disease burden.

### 6.4. Radiation Therapy

Given (a) the high proportion of patients with MPNST who also have NF1 and (b) the heightened radiation sensitivity of patients with NF1, radiation therapy should be used with caution in the context of MPNSTs. Radiation itself is a well-documented risk factor for MPNST (and other secondary malignancies) in patients with NF1 [75]. The median cumulative radiation doses needed for therapeutic efficacy are also quite high–often well in excess of 50 Gray (Gy) [63,76]. The literature generally suggests that radiotherapy is most useful in patients with large (e.g., >5 cm), high-grade tumors, and/or those with positive margins at resection [63,76,77]. It appears that the risk of local recurrence is reduced, but with an unclear benefit upon actual survival [76,77]. Moreover, radiotherapy timing (pre-operative versus post-operative) does not appear to have a statistical effect on the ability to achieve local control [78,79]. However, reasonable theoretical arguments can be made for either approach. The recent COG study ARST0332 included arms with both approaches, based on risk group assignment [42]. Patients who had undergone initial gross total resection subsequently underwent adjuvant radiotherapy (Arm B) or chemo-radiotherapy (Arm C) using 55.8 Gy (31 fractions of 1.8 Gy). Conversely, those with unresected or metastatic disease received both neoadjuvant and adjuvant chemo-radiotherapy (Arm D). This approach used 45 Gy (25 fractions of 1.8 Gy) neoadjuvant radiotherapy to the primary site, followed by resection and adjuvant radiotherapy with the dose determined by the success of resection–10.8 Gy (6 fractions of 1.8 Gy) for microscopic residual disease R1 (cumulative dose 55. 8 Gy) or 19.8 Gy (11 fractions of 1.8 Gy) for gross residual disease/no resection (cumulative dose 64.8 Gy) [42].

## 7. Conclusions

The MPNST treatment philosophy can therefore be summarized as thus: maximal surgical resection, potentially in combination with neoadjuvant chemotherapy or radiotherapy to render an initially inoperable tumor operable, followed by adjuvant chemotherapy or radiotherapy when the margins of resection are positive or metastatic disease exists.

## Figures and Tables

**Table 1:** National Institutes of Health diagnostic criteria for neurofibromatosis type 1 [26].

In an individual who does not have a parent diagnosed with NF1, two or more are required to make a diagnosis of NF1:
Pre-pubertal: ≥6 café-au-lait macules > 5 mm in diameter Post-pubertal: ≥6 café-au-lait macules > 15 mm in diameter
Axillary or inguinal freckling
≥2 neurofibromas of any type, OR ≥ 1 plexiform neurofibroma
Optic pathway glioma
≥2 Lisch nodules, OR ≥ 2 choroidal abnormalities
Presence of a distinctive osseous lesion: sphenoid dysplasia OR anterolateral bowing of the tibia OR pseudarthrosis of a long bone
Presence of a heterozygous pathogenic NF1 variant in apparently unaffected tissue (such as white blood cells), with a variant allele fraction of ≥50%
If an individual has a parent who meets the above diagnostic criteria, a diagnosis of NF1 may be made in that individual if one or more of the above criteria are present.

## Data Availability

Not applicable, the study did not report any data.
